# Partial N‐acetyl glutamate synthase deficiency presenting as postpartum hyperammonemia: Diagnosis and subsequent pregnancy management

**DOI:** 10.1002/jmd2.12388

**Published:** 2023-09-07

**Authors:** Lea Abou Haidar, Panayotis Pachnis, Garrett K. Gotway, Min Ni, Ralph J. DeBerardinis, Markey C. McNutt

**Affiliations:** ^1^ Children's Medical Center Research Institute The University of Texas Southwestern Medical Center Dallas Texas USA; ^2^ Howard Hughes Medical Institute The University of Texas Southwestern Medical Center Dallas Texas USA; ^3^ Department of Pediatrics The University of Texas Southwestern Medical Center Dallas Texas USA; ^4^ Eugene McDermott Center for Human Growth and Development The University of Texas Southwestern Medical Center Dallas Texas USA; ^5^ Department of Internal Medicine The University of Texas Southwestern Medical Center Dallas Texas USA

**Keywords:** carglumic acid, hyperammonemia, lactation, N‐acetyl glutamate synthase deficiency, pregnancy, urea cycle disorders

## Abstract

N‐acetyl glutamate synthase (NAGS) deficiency (OMIM #: 237310) is a rare urea cycle disorder that usually presents early in life with hyperammonemia. NAGS catalyzes the synthesis of N‐acetyl glutamate (NAG) which functions as an activator of the carbamoyl phosphate synthetase‐1 mediated conversion of ammonia to carbamoyl phosphate. The absence of NAG results in a proximal urea cycle disorder which can result in severe neurologic sequelae secondary to hyperammonemia and even death. Unlike the other urea cycle disorders, a specific pharmacological treatment for NAGS deficiency exists in the form of carglumic acid, an analog of NAG. Here we present a 29‐year‐old previously healthy female who presented with hyperammonemia and obtundation just after the birth of her first child. Exome sequencing revealed two novel variants in the *NAGS* gene, and plasma metabolomics revealed extremely low levels of NAG. Carglumic acid treatment led to prompt resolution of her biochemical abnormalities and symptoms. She tolerated two subsequent pregnancies, 2 years and 6 years after her initial presentation, while taking carglumic acid, and breastfed her third child, all without complications in the mother or children. This case report emphasizes the importance of considering urea cycle disorders in previously‐healthy adults presenting with neurological symptoms during periods of metabolic stress, including the postpartum period. It also highlights the efficacious and safe use of carglumic acid during pregnancy and while breastfeeding.

**Synopsis**

NAGS deficiency in a previously‐healthy postpartum woman treated with carglumic acid during subsequent pregnancies and breastfeeding.

## 1 INTRODUCTION

The urea cycle converts ammonia into urea which is then excreted in the urine.^1^ In patients with urea cycle disorders (UCD), ammonia levels can rise resulting in catastrophic neurologic sequelae.^2^ The conversion of ammonia to carbamoyl phosphate, catalyzed by carbamoyl phosphate synthetase‐1 (CPS‐1), is the rate‐limiting step of the urea cycle. CPS‐1 requires an allosteric activator, N‐acetyl glutamate (NAG), which is synthesized from glutamate and acetyl‐CoA by NAG synthase (NAGS). NAGS deficiency is one of the rarest urea cycle disorders, with only 98 cases reported by 2020.^3^ Several pathogenic variants in *NAGS* have been identified.^4^ Most patients with NAGS deficiency present early in life, although a few cases of later onset have also been reported.^4^ The classic presentation consists of symptoms typical of hyperammonemia such as vomiting, lethargy, altered consciousness, disorientation, ataxia, and food intolerance.^4^ NAGS deficiency can be treated with carglumic acid, a bioavailable analog of NAG that has been shown to activate the urea cycle. Here we present a previously healthy 29‐year‐old female with two novel variants in the *NAGS* gene, diagnosed with NAGS deficiency after an episode of hyperammonemia in the postpartum period. The safety of carglumic acid use during pregnancy and breastfeeding has not been evaluated. Here we present the safe and efficacious use of carglumic acid treatment in a patient with NAGS deficiency during two pregnancies and while breastfeeding.

## 2 METHODS

The patient and her parents were enrolled in an observational clinical research study (NCT02650622) approved by the Institutional Review Board (IRB) at the University of Texas Southwestern. Informed consent and blood samples were obtained from the patient and her parents.

Research‐based targeted metabolomics using liquid chromatography‐tandem mass spectroscopy (LC–MS/MS) was used to evaluate urea cycle intermediates. Patient plasma samples were collected before and after carglumic acid treatment and the control plasma samples were obtained from 24 healthy controls for targeted metabolomics (not fasting and not BMI, age, or sex matched). 50 μL of plasma was added to 950 μL of ice‐cold methanol/water 80% (vol/vol) and then dried in a SpeedVac concentrator (Thermo Savant) for 5–7 h. The metabolite pellets obtained for plasma samples were applied to targeted metabolite profiling using a LC–MS/MS approach using an AB QTRAP 5500 liquid chromatography/triple quadrupole mass spectrometer (Applied Biosystems SCIEX) with an injection volume of 20 μL. Chromatogram review and peak area integration were performed using MultiQuant software version 2.1 (Applied Biosystems SCIEX).

Research‐based whole exome sequencing (WES) of the patient and her parents was used to identify potential causative genetic variants. For DNA sequencing, the genomic DNA of the proband and both parents was sequenced using a SureSelect Human All Exon V4 kit (Agilent Technologies, Santa Clara, CA) for hybridization and capture. Paired‐end sequencing (150 base pairs) was performed using HiSeq2500 sequencing system (Illumina). Sequences were aligned to the human reference genome b37, and variants were called using the Genome Analysis Toolkit (GATK) HaplotypeCaller. Variants were filtered and identified using VarSeq (Golden Helix, Inc.). Variants were confirmed by Sanger DNA sequencing.

Variants were verified by clinical sequencing in a Clinical Laboratory Improvement Amendments Act of 1988 (CLIA) certified laboratory (GeneDx, Gaithersburg, MD). No pathogenic or likely pathogenic variants were detected in any other genes associated with primary or secondary causes of hyperammonemia.

## 3 CASE PRESENTATION

A previously healthy 29‐year‐old female presented with acute confusion and ataxia 5 days after the birth of her first child. Her pregnancy and vaginal delivery were uncomplicated. On evaluation, she was found to have an ammonia level of 167 μmol/L (Reference: <40 μmol/L). She has a past medical history of alopecia areata since childhood. She is a non‐smoker with no history of drug use or alcohol abuse. She did not previously have any unexplained vomiting episodes, poor appetite, food refusal/aversion, or other symptoms that would be consistent with a urea cycle defect. Family history was negative for genetic disorders, bleeding disorders, liver problems, history of cancer, or any autoimmune disease. No similar case presentations or metabolic disorders have been reported in any of her family members.

The patient was treated in the intensive care unit with an intravenous nitrogen scavenger and a dextrose infusion, however her ammonia levels continued to increase, peaking at 217 μmol/L. She thus underwent hemodialysis and continuous renal replacement therapy which then reduced her ammonia levels to 67 μmol/L. During dialysis, she developed hemolytic anemia and thus underwent three rounds of plasma exchange and received four units of packed red blood cells. Urine analysis showed glucosuria, ketonuria, hematuria, bilirubinuria, and hyaline casts, and she had a negative toxicology screen.

During the hyperammonemic episode, plasma amino acid analysis demonstrated elevated glutamate, glutamine, and lysine with low‐normal citrulline. No orotic acid or other organic acids were detected in her urine. On abdominal ultrasound, the liver was enlarged, and subsequent histology demonstrated scattered mega mitochondria and diffuse hepatocyte cytoplasmic glycogen excess.

**FIGURE 1:**
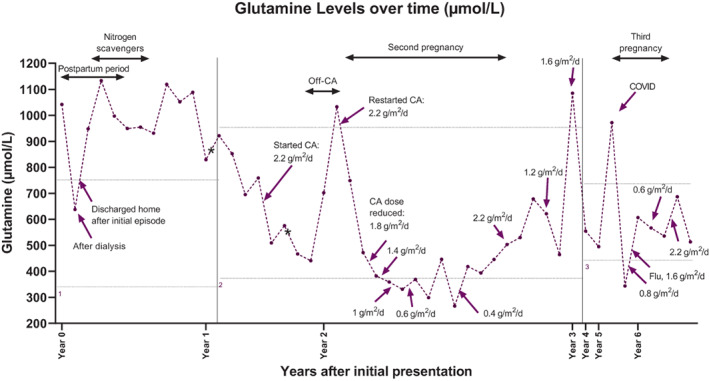
Plasma glutamine levels. Patient glutamine levels are shown over time. Carglumic acid dose, treatment adjustments, and major events are displayed during that period. Year 0 denotes the patient's initial episode. Glutamine levels used for longitudinal monitoring were highly elevated at initial presentation and fell with dialysis. As an outpatient, levels were consistently elevated regardless of nitrogen scavenger therapy. Levels fell precipitously with carglumic acid treatment, rose quickly with discontinuation of carglumic acid, but fell again rapidly with re‐initiation, remaining within the normal range on average throughout both pregnancies. Three different clinical labs were used over the course of monitoring (indicated by 1, 2, or 3) and each lab had different reference values. CA, carglumic acid; first asterisk (*), pre‐rx metabolomics sample date; second asterisk (*), post‐rx metabolomics sample date. Glutamine upper and lower limit of reference ranges (horizontal dotted lines): 1: 332–754 μmol/L, 2: 371–957 μmol/L, 3: 428–747 μmol/L.

Because her presentation was highly suggestive of a urea cycle defect, she was treated with the nitrogen scavenger sodium phenylbutyrate (10 g/m^2^/d, divided into 3 doses) and a protein‐restricted diet of <0.5 g/kg. She continued to have persistent elevations in plasma glutamine on this regimen (Figure 1); however her essential amino acids were normal. Six days after admission, her neurologic status was normalized, and she was started on oral citrulline (3.8 g/m^2^/d, divided into 2–3 doses). Once her ammonia levels improved and remained stable, her diet was liberalized to 0.8 g/kg protein, and the nitrogen scavenger was discontinued because the patient could not tolerate the high pill burden.

Clinical genetic testing for hyperammonemia, UCD, transporter defects, and other disorders associated with elevated ammonia panel/Next Gen sequencing of 44 genes reported one variant of uncertain significance (VUS) in *NAGS* (NM_153006.2): c.1080G > C (p.Glu360Asp). Research based WES then revealed compound heterozygosity of novel variants of unknown significance in *NAGS* (NM_153006.2): c.1080G > C (p.Glu360Asp) and c.426 + 3 G > C at the splice donor site of the first intron. At that time, the clinical lab was re‐contacted and they confirmed the presence of a second VUS in *NAGS* (c.426 + 3 G > C) that wasn't reported on the initial clinical report due to initial misclassification of the variant as likely benign.

**FIGURE 2:**
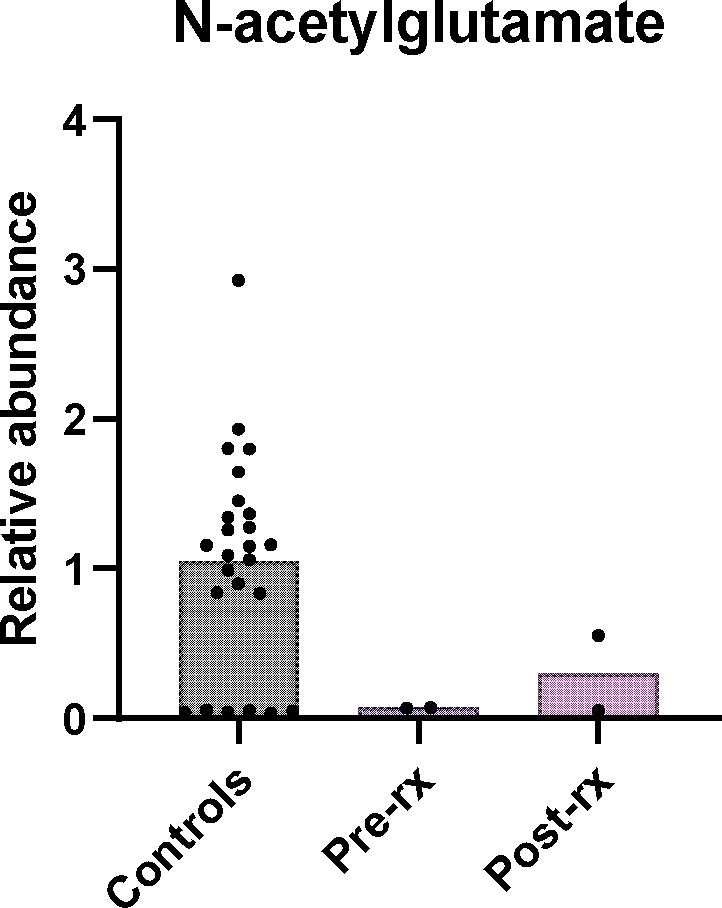
NAG from targeted metabolomics. NAG is shown with relative abundance normalized to the average of controls. Dots in the control column represent individual values for 24 different control samples. In several controls, NAG was not detected because the signal to noise ratio of the NAG peak was not above the baseline. Patient samples are shown before treatment with carglumic acid (pre‐rx) and 40 days (post‐rx) on treatment. The two dots on patient pre and post treatment samples represent replicate analysis of the same sample. NAG, N‐acetyl glutamate; pre‐rx: patient's samples before treatment with carglumic acid; post‐rx: patient's samples 40 days on treatment.

A research‐based metabolomics analysis of the patient's plasma revealed essentially absent NAG relative to controls (Figure 2). The clinical scenario, sequencing results and low NAG strongly implied a late‐onset form of NAGS deficiency. The patient was presumptively started on carglumic acid at a dose of 2.2 g/m^2^/d (patient body surface area at the time was 2 m^2^). Her glutamine and glutamate levels normalized rapidly with no further episodes of hyperammonemia. She experienced a few headaches shortly after starting or increasing the dose of carglumic acid, but otherwise tolerated it well.

In preparation for her second pregnancy, the patient was trialed off carglumic acid for 1 month, but her glutamine levels rose, prompting resumption of carglumic acid at the previous dose. Ammonia levels were not measured, and the patient did not report any clinical symptoms. The dose of carglumic acid was lowered iteratively based on glutamine levels and was 1.4 g/m^2^/d at the start of pregnancy. (The aim was to maintain the patient's glutamine levels to be lower than the upper limit of normal.) The dose continued to be reduced and was 0.4 g/m^2^/d by the end of the first trimester. Her amino acid levels were frequently monitored throughout the pregnancy to avoid abnormally low amino acid levels that might impair fetal growth. Due to the unknown risks of carglumic acid, fetal ultrasounds at 18–20 weeks, growth ultrasounds every 4 weeks, and weekly biophysical profiles starting at 32–34 weeks were scheduled (presented in Supplementary Files; Data S1 and S2). Recommended protein intake during pregnancy was 70 g/day. When amino acids (glutamine, glycine, ornithine) were low, recommended daily protein intake was increased to 80–90 g/day, however the patient was only able to maintain an intake of 75 g/day. The patient developed gestational hypertension (GHTN) but the pregnancy was otherwise uneventful. She was admitted for induction at 37 weeks due to GHTN and was started on 10% dextrose‐containing fluids which was maintained throughout labor until she was eating normally postpartum. Immediately after delivery, the carglumic acid dose was increased to 2.2 g/m^2^/d. The patient was monitored closely as an inpatient for 72 h. Plasma amino acid levels measured before delivery and 2 days postpartum were all within normal range. Ammonia levels were measured every 12 h after delivery for 24 h, and fasting ammonia levels were measured daily until postpartum day 3. These were also normal. One month after delivery, the carglumic acid dosage was decreased to 1.2 g/m^2^/d. Since the current evidence does not support breastfeeding while on carglumic acid, her second child was exclusively formula fed. The child's growth and development to age 4.25 years have been within normal limits (growth charts and prenatal measurements in supplementary data).

When the patient presented to the clinic 1 year postpartum, glutamine levels were elevated (1085 μmol/L, Figure 1). Her carglumic acid dosage was increased to 1.6 g/m^2^/d. She denied any episodes of metabolic decompensation during the previous 12 months. She remained stable on 1.6 g/m^2^/d for 2 years, after which she presented to the clinic 8 weeks pregnant with her third child. She underwent the same tapered decrease of her carglumic acid dose as during her second pregnancy, eventually going as low as 0.6 g/m^2^/d. Her protein intake recommendations were the same as during her second pregnancy (70 g/day). She was again diagnosed with GHTN and was admitted for induction at week 37. During this hospitalization, she experienced episodes of supraventricular tachycardia with palpitations and was prescribed metoprolol. She delivered via C‐section and was kept on beta‐blockers for 3 months postpartum and was also transiently treated with nifedipine for hypertension. Immediately after delivery, carglumic acid was increased to 2.2 g/m^2^/d. The patient decided to breastfeed her third child, despite the lack of safety data regarding taking this drug while breastfeeding. One month after delivery, her dose was decreased to 1 g/m^2^/d and she has remained stable on this dose. The third child's growth and development until 10.75 months old, have been within normal limits (growth charts and prenatal measurements in supplementary data).

## 4 DISCUSSION

NAGS deficiency is one of the rarest urea cycle disorders with an estimated incidence of 1:3500000–7000000.^3^ NAGS deficiency is inherited as an autosomal recessive disease that usually presents in infancy^5^, ^6^, ^7^, ^8^ with poor feeding, recurrent vomiting, tachypnea, lethargy, seizures, and coma.^4^ A few cases have been reported during childhood^9^, ^10^, ^11^, ^12^ and adulthood,^13^, ^14^, ^15^, ^16^, ^17^, ^18^ usually triggered by major stressors, and presenting with additional symptoms to those presenting in infancy, such as confusion, developmental delay, behavioral changes, and ataxia.^4^ Our patient presented with acute confusion at the age of 29, after giving birth to her first child. This highlights the importance of considering urea cycle disorders in adult patients presenting with acute confusion or other neurologic symptoms. Our patient's initial symptoms appeared after the birth of her first child. The postpartum period is a period of catabolic stress which can lead to metabolic decompensation in patients with urea cycle defects. Uterine involution, by which the uterus returns to its pre‐pregnancy state, relies on proteolysis and handling of a large nitrogenous load, within a short period. This can further overwhelm the system for the removal of nitrogen in patients with mild urea cycle defects leading to episodes of hyperammonemia.^19^, ^20^

After our patient's initial presentation, other causes of hyperammonemia including liver dysfunction, medications,^21^ infections (especially urease‐producing bacteria), and hematologic malignancies were excluded by the relevant workup. This prompted evaluation for genetic disorders of metabolism. Quantitative amino acid analysis showed elevated glutamine and alanine levels increasing the likelihood of a urea cycle disorder.^22^ Liver biopsy showed abnormal glycogen accumulation further supporting a diagnosis of an inherited metabolic disease such as a urea cycle defect^23^ or glycogen storage disease.^24^ Orotic acid levels, which are elevated in OTC (ornithine transcarbamylase) deficiency (OMIM #: 311250), were normal in our patient, making this particular urea cycle disorder less likely.^25^ Clinical genetic testing for single nucleotide variants or copy number variants in the *OTC* and other primary urea cycle genes was non‐diagnostic. This prompted us to perform WES which revealed two novel variants in the *NAGS* gene.

Several pathogenic variants have been reported in the *NAGS* gene,^13^ but none of these were identified. Our patient's WES demonstrated two novel variants in the *NAGS* (NM_153006.2) gene: 1. c.1080G > C (Glu360Asp) 2. c.426 + 3 G > C. The variants were confirmed in trans by parental testing. After learning of this patient's variants, our collaborators showed that the Glu360Asp variant (corresponding to Glu354Asp in mice) abolishes binding and activation of NAGS by l‐arginine.^26^ In mammals, activation of NAGS by l‐arginine is required for normal ureagenesis and so abrogation of this mechanism can result in defective ureagenesis and hyperammonemic episodes. In addition to genetic testing, we performed metabolomics on the patient's plasma. There are no clinical tests to measure NAG levels, but metabolomics reports the abundance of many metabolites without requiring a priori knowledge of the site of the defect. The low plasma NAG levels in the patient helped us interpret the functional impact of the variant in intron 1.

Carglumic acid, approved by the FDA in 2010, is the treatment of choice for NAGS deficiency.^27^ The initiation of carglumic acid resulted in a rapid correction of the patient's biochemical abnormalities. Carglumic acid is generally very well tolerated, but its use can still cause certain adverse reactions. For instance, 3 out of 13 patients taking carglumic acid experienced headaches as reported in the literature.^28^ Our patient experienced headaches when she first started carglumic acid therapy and when the dose was increased, however, her amino acid and ammonia levels were normal during those episodes. She has a history of migraines with similar presentations, so it is unclear if the headaches were adverse effects of carglumic acid.

There is currently no adequate data on the use of carglumic acid in pregnant women. Its use has been labeled as Category C in pregnancy because decreased survival and growth occurred in offspring born to animals receiving a dose approximately 38 times the maximum reported human dose.^28^

Most reported complications in pregnant patients with UCD occur in early pregnancy and postpartum due to several possible metabolic stressors such as hyperemesis gravidarum and malnutrition; there have been reports of undiagnosed UCD patients who developed fatal hyperammonemic encephalopathy during pregnancy.^19^ Untreated NAGS deficiency results in irreversible neurologic damage and death, thus women with NAGS deficiency must have strict disease control throughout pregnancy.^28^ At the time, a discussion was held between the patient and her care team, and despite all unknowns about the disease and its treatment during pregnancy, the patient opted to continue carglumic acid treatment throughout her pregnancy to avoid any possible decompensations in the perinatal period.

It is still unknown whether carglumic acid is safe to take while breastfeeding. Carglumic acid is excreted in rat milk, and neonatal rats nursed by mothers receiving carglumic acid were reported to have increased mortality and impairment of weight gain.^28^ Although studies have shown carglumic acid to be present in milk from treated rats, it is still unknown whether carglumic acid is present in human milk. There is no available data on the effects of carglumic acid on breastfed infants, and is thus not currently recommended. Weighing the risks and benefits, our patient chose to breastfeed her third child. To date, the infant has had no ill effects from this, suggesting that carglumic acid may be safe to take by NAGS‐deficient mothers who are wishing to breastfeed.

## 5 CONCLUSION

NAGS deficiency is a rare urea cycle disorder, often diagnosed in infants, that leads to the toxic accumulation of ammonia in the blood. We identified two novel variants in the NAGS gene of a female patient presenting with hyperammonemia after the delivery of her first child. Metabolomics analysis demonstrated reduced NAG levels, further supporting NAGS deficiency. The patient was successfully treated with carglumic acid throughout two further pregnancies and successfully breastfed her third child without any adverse outcomes. NAGS deficiency should be considered in the differential of unexplained cases of hyperammonemia in adults as late‐onset presentation is possible, and a safe and effective therapy is available in the form of carglumic acid. With further studies, we hope that carglumic acid treatment will be established to be a safe treatment during pregnancy and lactation in patients with NAGS deficiency.

## AUTHOR CONTRIBUTIONS

Lea Abou Haidar reviewed the literature and collected the patient data by chart reviews. Lea Abou Haidar, Panayotis Pachnis, Min Ni, Ralph J. DeBerardinis, Markey C. McNutt, and Garrett K. Gotway wrote and edited the manuscript. Lea Abou Haidar, Panayotis Pachnis, Min Ni, Markey C. McNutt, and Ralph J. DeBerardinis analyzed and interpreted the research data. Garrett K. Gotway and Markey C. McNutt obtained and interpreted clinical data, provided direct patient care and clinical expertise on urea cycle defects.

## CONFLICT OF INTEREST STATEMENT

Lea Abou Haidar, Panayotis Pachnis, Garrett K. Gotway, and Min Ni declare that they have no conflict of interest. Ralph J. DeBerardinis is a founder and advisor for Atavistik Bioscience. He is also a member of the Scientific Advisory Board for Agios Pharmaceuticals and Vida Ventures. Markey C. McNutt has received honoraria from Horizon Therapeutics, Biomarin Pharmaceuticals, Eton Pharmaceuticals, Acer Therapeutics, Ultragenyx, Applied Therapeutics, Jnana Therapeutics and Bridge Bio and been a site PI for clinical trials sponsored by Aeglea Biotherapeutics, Reneo Pharmaceuticals, PTC Therapeutics, Homology Medicines, Horizon Therapeutics, and Arcturus Therapeutics.

## ETHICS STATEMENT

The patient and her parents were enrolled in a study focused on developmental and metabolic anomalies entitled “Genetic Regulators of Metabolism and Development in Children”, approved by the Institutional Review Board (IRB) at The University of Texas Southwestern Medical Center (UTSW). Protocol number STU 112014‐001.

## INFORMED CONSENT

All procedures followed were in accordance with the ethical standards of the responsible committee on human experimentation (institutional and national) and with the Helsinki Declaration of 1975, as revised in 2000 (5). Informed written consent was obtained from the patient and her parents for being included in the study.

## Supporting information


**DATA S1:** Supporting Information.

**DATA S2:** Supporting Information.

## Data Availability

Data sharing is not applicable to this article as no new data were created or analyzed in this study.
