# Supplementary material for: Partial N‐acetyl glutamate synthase deficiency presenting as postpartum hyperammonemia: Diagnosis and subsequent pregnancy management
Source: JIMD Rep. 2023 Sep 7;64(6):403–9. doi: 10.1002/jmd2.12388 (PMC10623101; doi:10.1002/jmd2.12388)
Supplement: Supplementary file 1 — DATA S1: Supporting Information. [file JMD2-64-403-s001.docx]

Her second baby had an appropriate gestational age, normal heart rate (HR), and appropriate interval growth parameters with reassuring biophysical profiles during all fetal ultrasounds performed.

His weight was 380 grams (g) (55^th^ % for gestational age (GA)), and head circumference (HC) was 18.2 cm (50^th^ % for GA), and femoral length (FL) and humeral length (HL) which are indicative of height were 3.3 cm and 3.1 cm respectively, placing him at the 47^th^ and 45^th^ % for GA at the 20w 4d ultrasound.

At 24w1d, he was 738 g (69^th^ % for GA), HC was 22.8 cm (66^th^ % for GA), and FL was 4.6 cm (77^th^ % for GA).

At 28w4d, weight was 1613 g (93^d^ % for GA), HC was 28.5 cm (90^th^ % for GA), FL was 5.8 cm (79^th^ % for GA), and HL was 5.5 cm (> 95^th^ % for GA).

At 32w0d, weight was 2070 g (61^st^ % for GA), HC was 30.9cm (87^th^ % for GA), FL was 6.3 cm (59^th^ % for GA), and HL was 5.2 cm (24^th^ % for GA).

At 36w0d, weight was 3164 g (79^th^ % for GA), HC was 33.5 cm (88^th^ % for GA), and FL was 7.0 cm (49^th^ % for GA).

Growth charts for Stature and Weight for ages 2.5 years old until 4.25 years old are shown in the Supplementary Figure 1.

Supplementary Figures 1: Baby 2


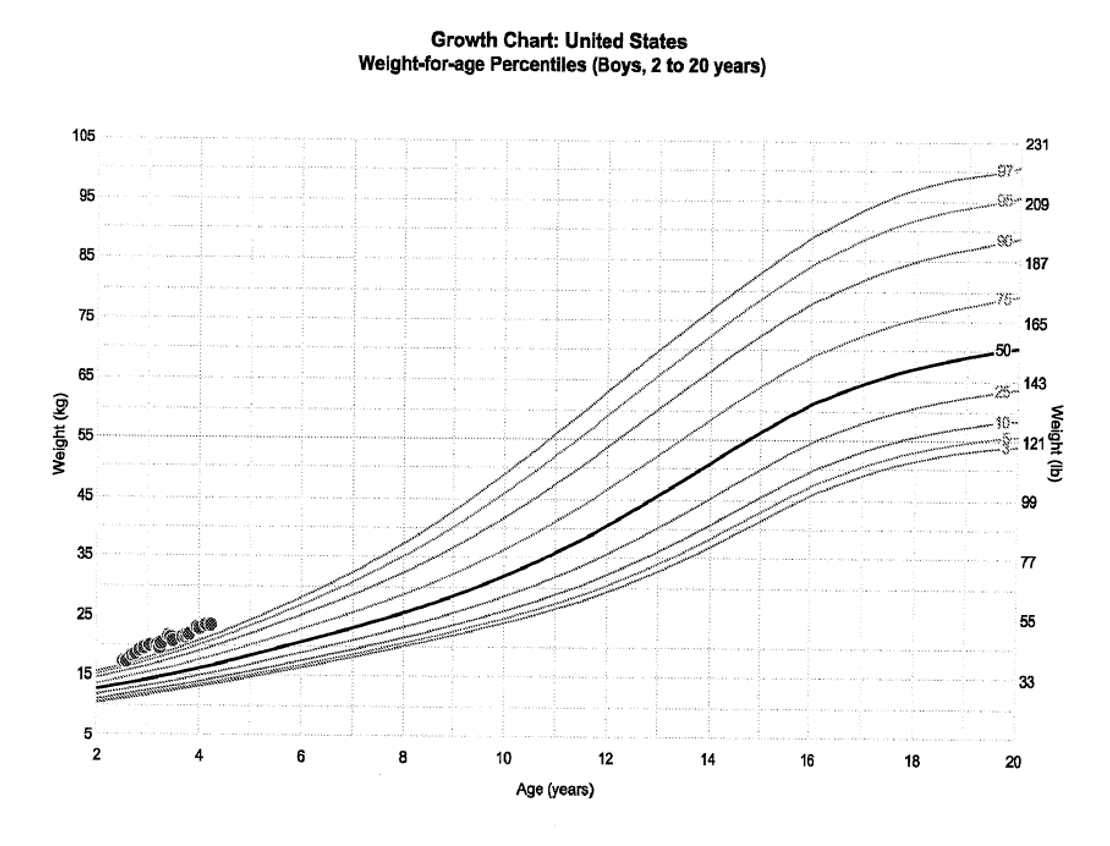


Supplementary Figure 1A


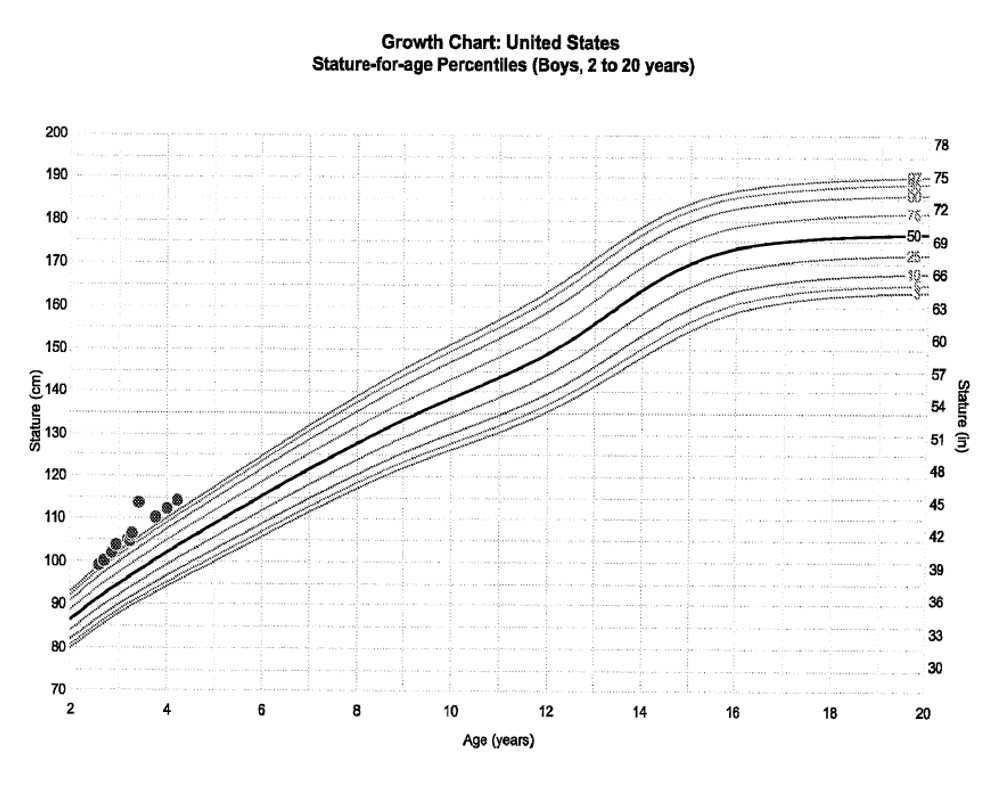


Supplementary Figure 1B

Her third baby had an appropriate gestational age, normal HR, and appropriate interval growth parameters and reassuring biophysical profiles during all fetal ultrasounds performed.

At 21w1d, weight was 431 g (66^th^ % for GA), HC of 18.3 cm (21^st^ % for GA), FL of 3.7 cm (58^th^ % for GA), and HL of 3.5 cm (65^th^ % for GA).

At 24w6d, weight was 821 g (70^th^ % for GA), HC was 22.3 cm (13^th^ % for GA), FL was 4.6 cm (52^nd^ % for GA) and HL was 4.2 cm (60^th^ % for GA).

At 28w6d, weight was 1417 g (64^th^ % for GA), HC was 26.7 cm (22^nd^ % for GA), FL was 5.9 cm (83^d^ % for GA) and HL was 5.1 cm (65^th^ % for GA).

At 32w6d, weight was 2371 g (81^st^ % for GA), HC was 31.0 cm (61^st^ % for GA), FL was 6.6 cm (65^th^ % for GA), and HL was 5.6 cm (46^th^ % for GA).

At 36w6d, weight was 3636 g (95^th^ % for GA), HC was 32.7 cm (25^th^ % for GA), FL was 7.5 cm (83^d^ % for GA), and HL was 6.4 cm (53^d^ % for GA).

Growth charts for Length, Weight, and HC for ages 0 months old until 10.75 months old are shown in the Supplementary Figure 2.

Supplementary Figures 2: Baby 3


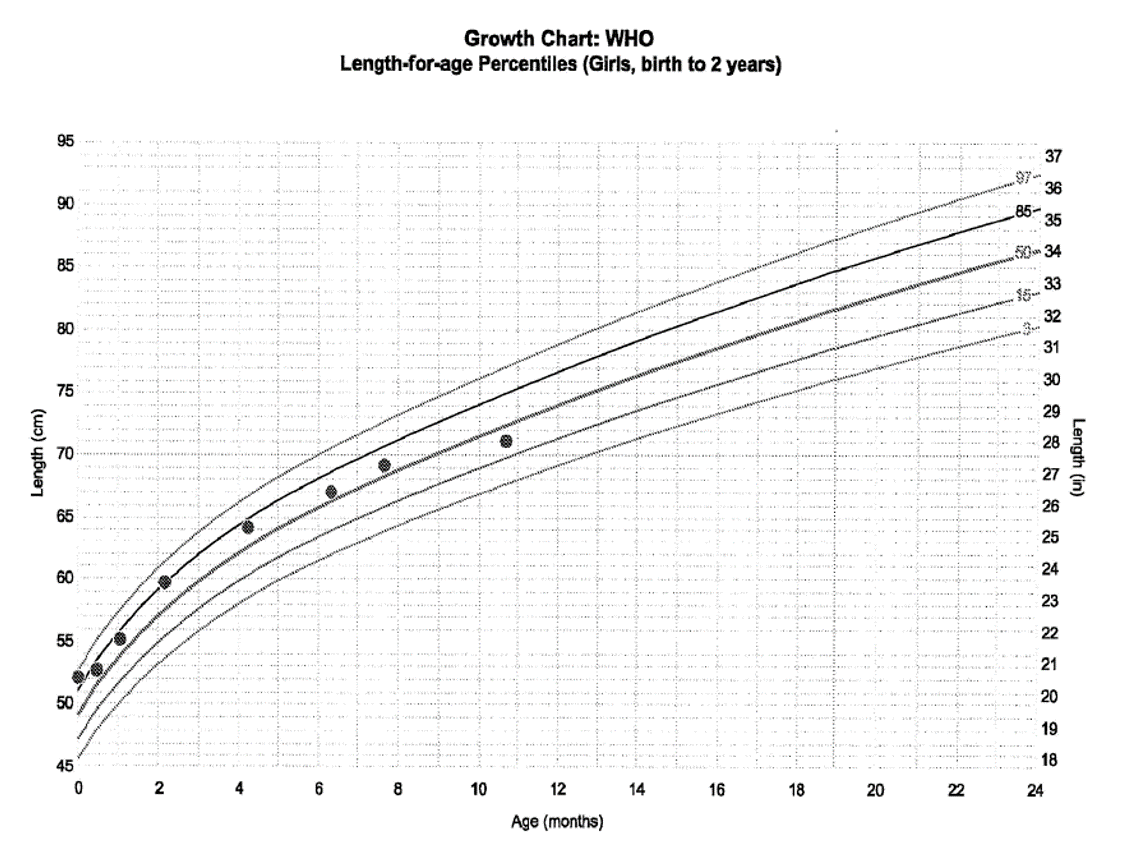


Supplementary Figure 2A


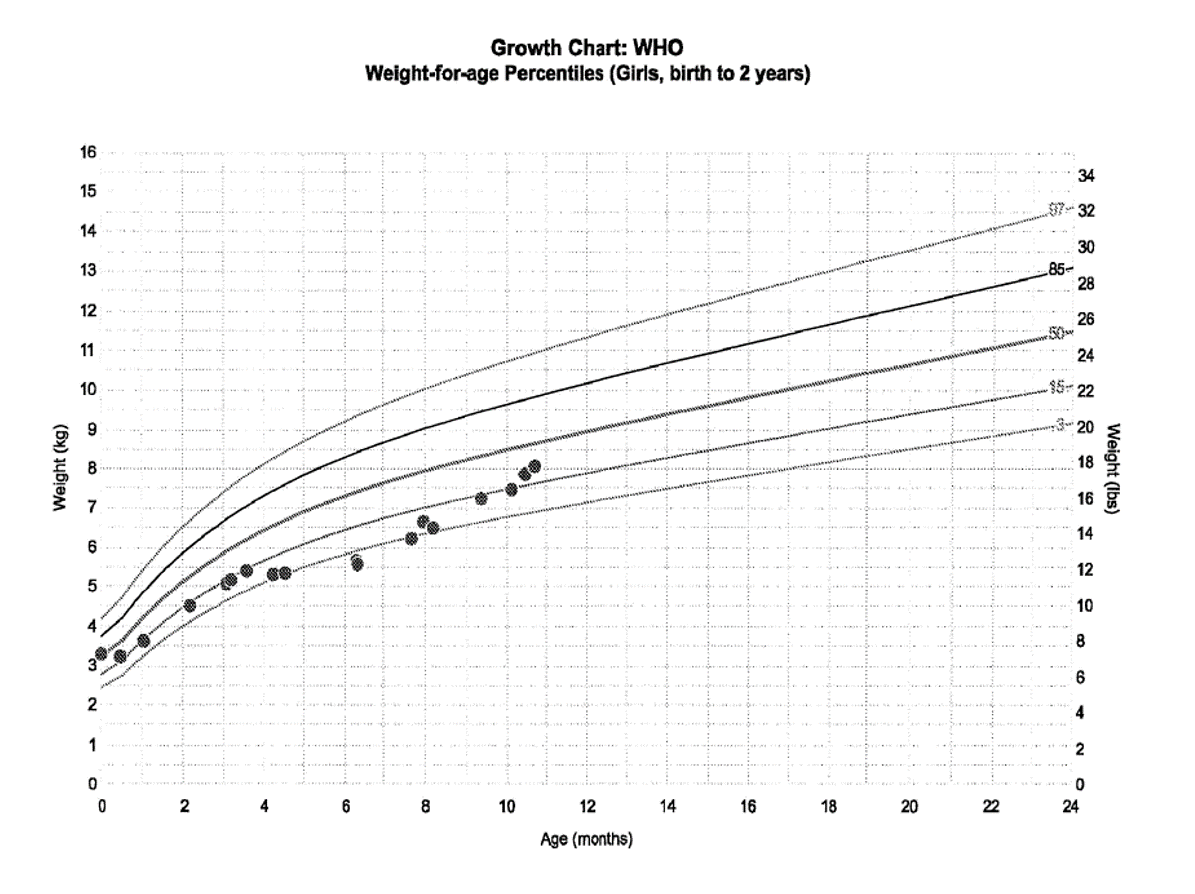


Supplementary Figure 2B


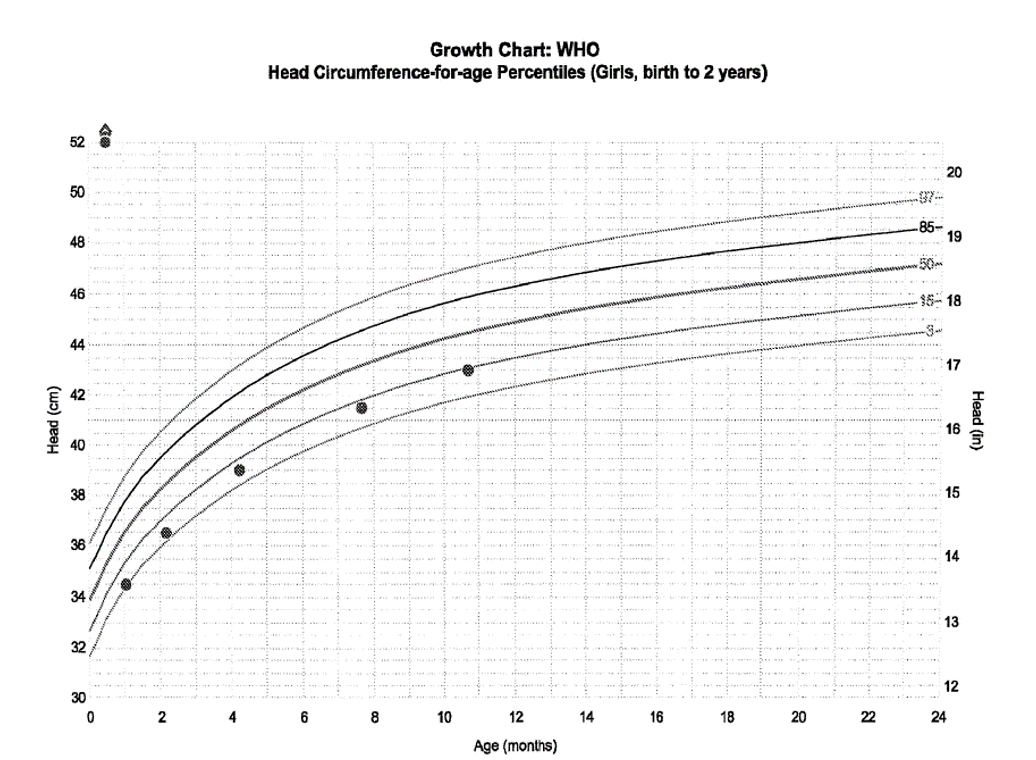


Supplementary Figure 2C
